# A review on the evolution of simulation-based training to help build a safer future

**DOI:** 10.1097/MD.0000000000029503

**Published:** 2022-06-24

**Authors:** Jared Bienstock, Albert Heuer

**Affiliations:** Rutgers School of Health Professions, Department of Interdisciplinary Studies, Newark, NJ.

**Keywords:** COVID-19, history of simulation, interprofessional teamwork, simulation-based training

## Abstract

Throughout history, simulation has been used to demonstrate various techniques, acquire skills, and maintain best practices in healthcare. Simulation has evolved significantly, primarily because of the extent to which it can enhance both clinical proficiency and patient care. Although simulation-based training (SBT) in healthcare has grown exponentially in the 21^st^ century, it has been around for centuries. This paper aims to reflect on the history and evolution of simulation in healthcare and review its current applications in order to provide a foundation for developing new applications for future expanded use.

## 1 Introduction

Simulation-based training (SBT) has been recognized to enhance quality and safety in many industries, including aviation and healthcare. Although the aerospace industry is relatively “young” compared to healthcare, SBT has become the standard in training pilots and other aviators. Hence, the aerospace industry has become one of the safest industries, despite the extraordinary inherent risks. In contrast, while SBT has been employed in healthcare for centuries, many quality and safety issues persist today. This is demonstrated by the fact that approximately one in every ten patients worldwide is harmed during their care in a hospital in high-income countries, where 50% is preventable.^[1]^ A further irony is that simulation has been underutilized in medical education or training, despite “ample evidence that the amount of preventable errors and harm from medical care makes admission to hospital many times more dangerous than air travel.”^[2]^

SBT use in healthcare has increased, its uptake has been slow, and an often insufficient macro-level strategic focus has limited our healthcare system's ability to reap full benefits. This is substantiated by some of the findings within the systematic review completed by Heuer et al. entitled Simulation-based Training Within Selected Allied Health Professions: An Evidence-Systematic review (2022). This systematic review identified vast differences in the use of simulations, pointed to the underutilization of SBT by several health disciplines and emphasized the importance of more rapid and widespread use.^[3]^ However, most forms of expansion, including those related to simulation, require a base from which to launch. In essence, future advances in an area of interest can often be facilitated by understanding past and current state affairs. Applying this premise to SBT, in addition to providing a historical perspective, this narrative aims to provide such a base by reflecting on the historical evolution of medical simulations to the present day, as a launching point from which new discoveries and expanded use of SBT can be realized in the future.

## 2 Methods

We explored articles on PubMed and other databases related to SBT and its history, past, present, and future states. We utilized key terms and phases including “history of simulation, simulation-based training, interprofessional teamwork,” and “the future of simulation-based training.” To explore a more recent development that has impacted SBT, we also searched for terms related to the impact of Coronavirus disease 2019 (COVID-19) on the use of SBT. Both authors agreed to the selected references if they comprised relevant information that met the aim of this review.

## 3 The past history of simulation

SBT was utilized thousands of years ago to “develop competence and confidence in students and train health professionals” for real-life situations.^[4]^ Stone carvings of the human form were discovered through Eurasia, dating back to 24,000–22,000 BC. In 1900–1600 BC Babylonia, clay livers were discovered and believed to be used to determine the outcome of an illness.^[2]^ In the 6^th^ century BC, Lau Tzu wrote about automation machines, which he described as being made of wood, leather, and glue, could walk, sing, gesture, and wink.^[2]^ Badash et al.^[5]^ described surgical simulators used more than 2500 years ago, which could bleed, leak fluid, and were made from materials to mimic human bodies.

Hippocrates, the father of medicine (460–367 BC), suggests that simulation should focus on practical training of health professionals in his *Aphorismi*, *Ars longa, vita brevis*.^[2]^ Between 385 and 322 BC, the Greek philosopher Aristotle taught the importance of repetition in developing expertise and the necessity of feedback and guidance in his *Nichomachean Ethics*, emphasizing the necessity for training models.^[2]^ The oldest written evidence of simulation in healthcare education was the *Sushruta Samhita*, which documented how to develop and utilize simulators for surgical training skills by using wooden objects to manage wounds.^[2]^

During the Song Dynasty in China (960–1279 CE), physicians used life-sized bronze statues to teach surface anatomy and acupuncture. These simulators had organs and holes for needle insertion. The Ch’ing Dynasty (1644–1912 CE) used female figurines carved out of ivory to meet the needs of male physicians who were not allowed to examine women.^[4]^

At other points in history, human dissections were not accepted and made illegal by the Catholic Church. In 16^th^ century Europe, Andrea Vesalio sparked interest in studying anatomy. Since acquiring dissected material was not easy, difficult to preserve, and illegal, wax figures were made, the first one traced back to 1598 CE.^[4]^ The Catholic Church only permitted wax body parts as part of the simulation, rather than human dissections.^[2]^ In 17^th^ and 18^th^-century Europe, anatomical figures were widely used to teach medical students and doctors.^[4]^

The first doctoral thesis on simulation was written in the 18^th^ century by Georg Heinrich von Langsdorf, *Phantasmatum Sive Machinarum Ad Artis Obstetriciae Exercitia Fascientium Vulgo Fantome Dicratum Brevis Historia*. The title of this text means “A short account of likeness or devices for practicing obstetric skills, also called phantoms.”^[2]^ The word phantom refers to manikin, which assists obstetricians with teaching delivery techniques.^[6]^ The middle of the 18^th^-century revealed what is believed to be the first example of a learning need, indicating the need for simulation training. A surgeon named Giovanni Antonio Galli designed a glass uterus with a fetus to train midwives and surgeons during childbirth, which is the first documented evidence of SBT.^[4]^ The 18^th^ century also documented many attempts to develop machinery that could simulate cardiovascular physiology.

Although there has been growth in medical simulation throughout history, Owen^[4]^ explains that the 20^th^ century was not expedient in simulation advancements, referring to this period as the “dark age” for simulation. This period depicts the time at which the patients were used to practice skills. Despite this claim by Owen,^[4]^ Aebersold^[7]^ describes several advances made in the 20^th^ century. For example, in 1911, Martha Jenkins Chase, a doll maker, made a doll to train nurses on dressing, turning, administering medication, and transferring patients. In the 1940s, the US Army used manikins to teach medical corpsmen medical techniques.^[8]^

In the 1960s, Resusci Anne was developed, which revolutionized the ability to train at a low-cost yet effective model. Around the same time, a simulator known as SimOne was developed, which is a sophisticated manikin with the ability to breathe, has a heartbeat, pulse, blood pressure, mouth movements, blink, and response to drugs. During the 1960s–1970s, the Harvey simulator was developed, which is a cardiology patient simulator with a hybrid task-trainer and computer-enhanced manikin.^[9]^ In the 1980s, a high-fidelity (HF) simulation was resurrected. Gaba developed a comprehensive anesthesia simulation environment (CASE), and Michael Good and Gravenstein developed the Gainesville anesthesia simulator (GAS).^[10]^ Gaba assisted in making computerized manikins. These HF simulators were the basis for the present-day simulators. These transformations, which were simulated in the late 20^th^ century, led to the growth of HF simulations.^[10]^

**Figure 1:**
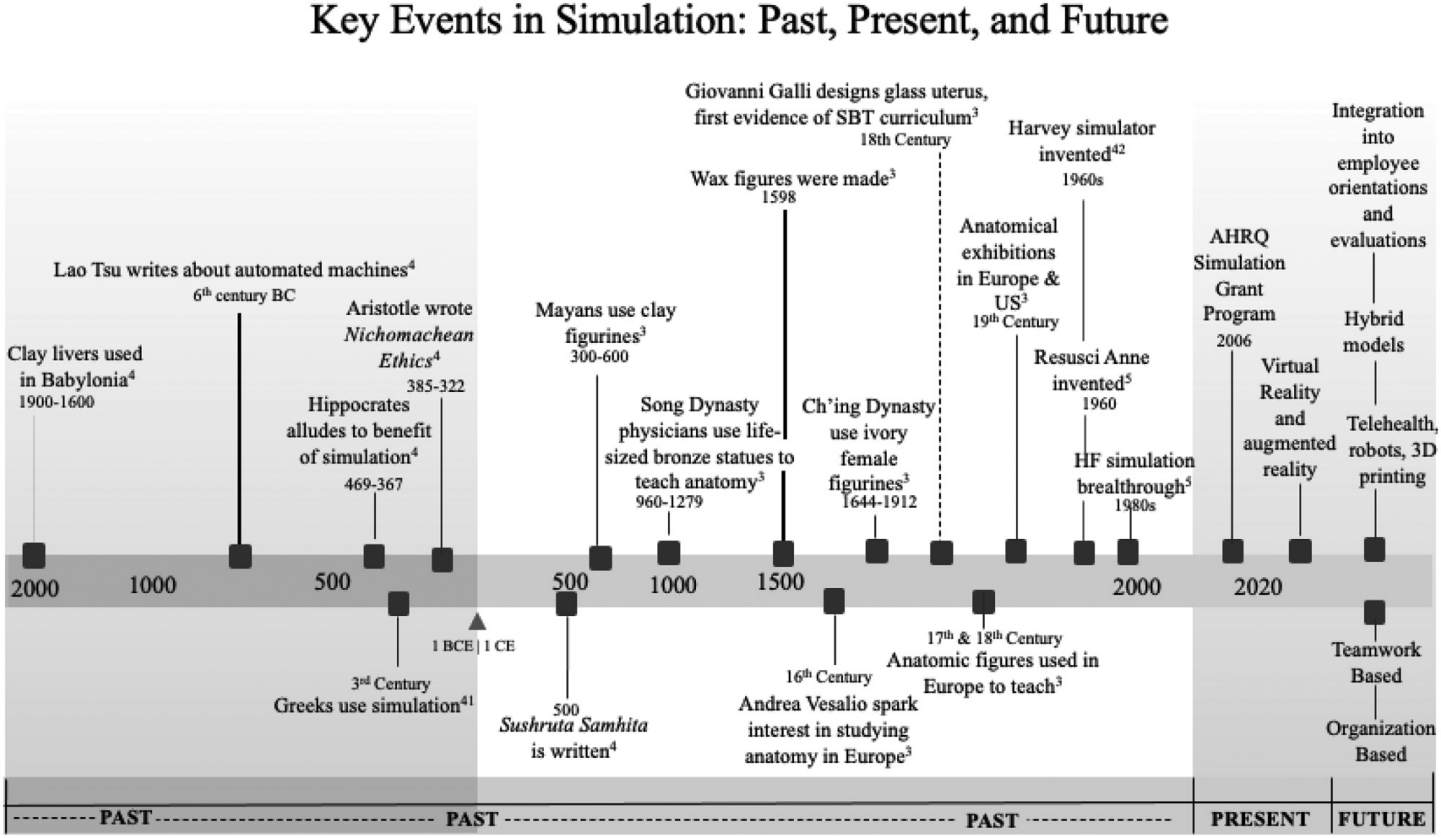
Timeline of the history of simulation. Timeline of the key events in the history and evolution of simulation for the past, present, and future.

Early adopters of simulation understood its importance and criticality in improving care. There was an understanding that it assisted in developing skills without putting patients at risk. Skills can be practiced as much as possible, ultimately allowing practitioners to become experts in their trade.^[4]^ Simulators have undergone extensive evolution from anatomical figures made of clay and others made of wax, and ultimately computerized simulators. Despite the differences in material and technological capabilities, they all provided a foundation for building, a century after century, leading to their widespread adoption and effectiveness for future users to help improve patient care and safety. Figure 1 provides a timeline of the key events in the history and evolution of the simulation.

## 4 Present day use of simulation

It is believed that the modern era of simulation originated in the second half of the 20^th^ century. Within “the last two decades, there has been an exponential rise in the development, application, and general awareness of simulation use in the healthcare industry.”^[11]^ Modern-day simulation is an integral part of education and training in some institutions. However, simulation centers with dedicated equipment and trained personnel have not become standard.^[12]^

Despite significant strides in healthcare simulation throughout history, many practical skills are being taught at the bedside of actual patients, exposing them to risks associated with learners acquiring new skills.^[11]^ Furthermore, there is still an ill-defined role for simulation in healthcare arenas. It is only within the last few decades that most medical disciplines are developing a collective vision for what simulation's role is, how to train, educate, rehearse procedures, maintain competency, and advance skillsets. The early opposition that simulation has met in the healthcare industry is fading away with advancing technologies and advanced training methods. Extensive work has been done to “demonstrate and make simulation a rigorous tool for training and assessment.”^[11]^ For decades ago, simulation seemed relevant only for improving psychomotor skills, and the modern era has expanded SBT to include improving teamwork, communication, and enhancing nontechnical human factors.

Clinical expertise is insufficient to claim competence in the healthcare environment. This is one reason why SBT engagement applies to physicians as well. Simulations have been adopted throughout medicine for physicians across many disciplines. Anesthesiology, pulmonology, cardiology, cardiac surgery, and many other disciplines use simulation to build and maintain skills as well as assess competency. The vast complexities of patient care require physicians and other health professionals to not only master specific knowledge and procedural skills but also “to effectively communicate with patients, relatives, and other health care providers and also to coordinate a variety of patient care activities.”^[13]^ Often, the leader of a multidisciplinary team, physicians must master their practice to manage a team effectively.

Simulation was slow to be adopted by nursing in the 1990s. Since then, simulations have proven to be significant in improving performance, including being used in conducting a root-cause analysis to address adverse events. Initially, many nursing educators were reluctant to adopt simulation because of the perception that simulation technology requires expertise to manage.^[11]^ However, many hospitals have begun to incorporate SBT into orientation processes and competencies for nurses and other health professionals. Today, nursing simulators feature vital signs and physiological responses using computer programming. Nursing training has been reported to use screen-based simulators such as Second Life©, an interactive virtual web-based environment used for role-playing involving crisis scenarios. Scenarios are created to manage patients, use clinical judgment, and interventions. Standardized patients, manikins, and part-task trainers can all be used in low-to high-fidelity simulations for nurses.^[11]^

Many allied health professionals use simulations to develop clinical skills. In particular, Heuer et al^[3]^ demonstrated that paramedics, emergency medical technicians (EMTs), and respiratory therapists are especially heavy users of SBT in clinical training. This may be related to a combination of factors, including a high-acuity environment in which they often function and a comparatively short (undergraduate or certificate) education preparation, placing a premium on the need for this type of training for practitioners in these professions. Not surprisingly, the types of simulations used in these allied health professions include procedures related to cardiopulmonary resuscitation and emergency airway management, which are error-prone and disproportionately result in patient harm.

## 5 Use of simulation in interprofessional teams

Traditionally, healthcare professionals have been trained within their professional silos and are rarely trained together. A significant step in the evolution of simulation is heightened use in building teamwork and interprofessional collaboration, which are critical to clinical practice and enhance patient outcomes.^[14]^ Medical errors, which result in death or injury, often stem from communication breakdowns and poor-quality teamwork. Therefore, participation in teamwork training simulation can enable healthcare professionals and organizations to improve clinical performance and patient outcomes.^[15]^ This type of approach to SBT is known as interprofessional teamwork training or simulation-based team training (SBTT).^[16]^

SBTT in healthcare found its roots in crisis resource management (CRM), which was initially used in the aviation industry in the 1970s. CRM is a system created to optimize equipment, procedures, and people with the goal of patient safety. This is a strategic approach to improving teamwork and communication skills. CRM focuses on individual and team behaviors in ordinary and crisis scenarios while simultaneously concentrating on decision-making skills, interpersonal behavior, and team management.^[11]^

**Figure 2:**
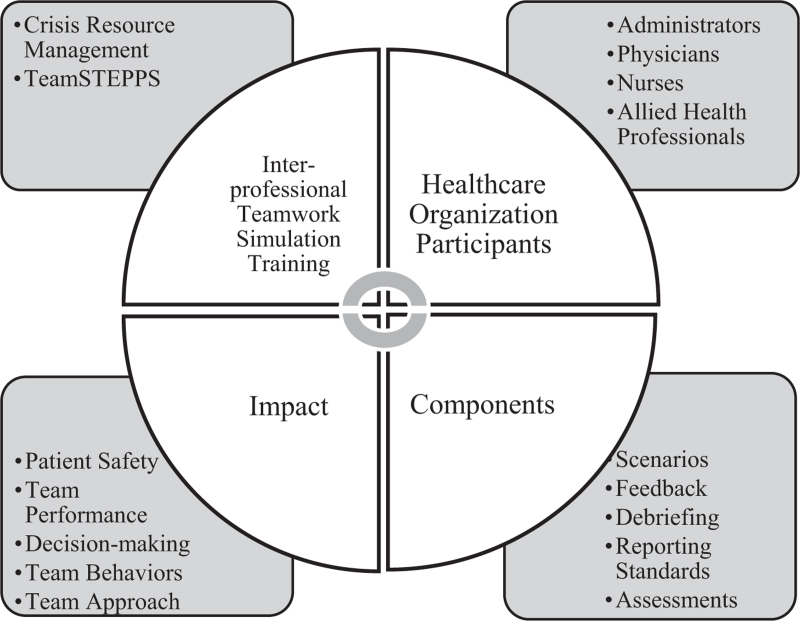
Interrelationship between SBTT and its components. The interrelationship between SBTT and its components are comprehensive and synergistic.

There are several key components of SBTT: well-designed scenarios, feedback, debriefing, reporting standards, and validation through outcome assessment. The Institute of Medicine (IOM) emphasizes the importance of interprofessional teamwork in enhancing patient care through improved collaboration and communication. Communication failures are among the top contributing factors to adverse events and deaths.^[17]^ Sentinel events in healthcare often do not originate from a single root cause. Instead, they often involve a combination of human factors and system failure.^[18]^ The proper use of SBTT techniques can enhance the delivery of patient-centered care.^[19]^ “Team training, including the use of simulations, for licensed healthcare professionals has been associated with improvements in patient outcomes and a decrease in adverse events.”^[20]^ SBTT is paramount for present-day and future patient safety and teamwork building. According to Boet et al,^[14]^ “high-quality interprofessional care has been shown to contribute to improving staff morale, greater patient satisfaction, and may improve patient safety.” Thus, the SBTT strives to improve the multidisciplinary team approach to medical scenarios. SBT has gained prominence in interprofessional collaboration predominantly in the late 1990s via crisis resource management and team strategies and tools to enhance performance and patient safety (TeamSTEPPS) in the early 2000s. The relationship between SBTT and its components is illustrated in Figure 2.

Another method of teamwork building is known as Team Strategies and Tools to Enhance Performance and Patient Safety (TeamSTEPPS), which evolved out of CRM.^[11]^ The Agency for Healthcare Research and Quality (AHRQ) developed TeamSTEPPS to develop effective teamwork in the healthcare field via evidence-based simulation based on teamwork training.^[21]^ According to the AHRQ, “as a training approach, simulation offers an opportunity to teach and engage health care professionals in a manner far superior to traditional methods of lecture and demonstration.”^[22]^ These two strategic approaches are essential tools for managing SBTT, as they are designed to promote and improve patient safety and improve team performance.

## 6 Simulations and COVID-19

Although much progress has been made with SBT, there are gaps in our progress in this arena that have been clearly identified due to COVID-19. As a result of the pandemic, there was widespread discontinuation of clinical education for our health professions, which reduced or outright eliminated opportunities for students and practicing clinicians to build their hands-on clinical proficiency. This resulted in skills gaps that are now only beginning to be appreciated. According to Bentata,^[23]^ the repercussions of COVID-19 led to “the interruption of courses in medical schools and/or hospital training rotations, the introduction of teaching exclusively online, postponement of examinations, and of the new academic year.”

A variety of studies have illustrated the impact of COVID-19 on healthcare training. Although temporarily suspending education and training was intended to protect the population and help mitigate the spread of COVID-19, many healthcare students and professionals fell victim to a training gap. Skill deterioration was a result of the pandemic. Skill deterioration is defined as the “loss of acquired skills after a period of non-use, which has been found to increase as the nonpractice interval lengthens.”^[24]^ One study by Marasco et al^[25]^ found that the interruption in elective cases made it challenging to maintain high standards of training young gastroenterologists in Europe due to a training gap. Training gaps were found due to a lack of elective cases and non-involvement of mentors in activities due to the need for COVID-19. This pandemic has revealed other existing gaps. Infection control protocols and skills that were initially thought to be common practice at the bedside were inadequate to deal with the airborne spread of COVID-19. In a survey completed by Mishra et al,^[26]^ ophthalmic trainees reported that COVID-19 negatively impacted their surgical training. A survey by Ferrara et al^[27]^ showed that more than 70% of trainees reported more than a 50% reduction in clinical activity and a 75% reduction in surgical activity due to COVID-19.

SBT is a means to build clinical skills and help bridge gaps created by the pandemic. In the presence of the COVID-19 pandemic, simulations have enabled clinicians to practice the proper use of personal protective equipment (PPE), other infection control techniques, and develop skills in new and expanded roles, including processing equipment and assisting with prone positioning of infected patients with respiratory failure. A tertiary care center reported using rapid-cycle in situ simulation (ISS) to enhance departmental preparedness for COVID-19.^[28]^ This simulation model aims to improve teamwork, identify latent safety threats, address informing processes, and guide clinical protocols and care. Participants reported that concerns were addressed after participating in the simulation and ultimately reinforced the significance that rapid-cycle ISS training can meet the need to improve preparedness during pandemics.^[28]^

## 7 The future of simulation

Although simulation has come a long way, especially over the past few decades, there is still more work to be done to derive full benefits. According to one survey, “most simulation centers were positive on the future of simulation, predicting a 55% growth in the next 5 years….”^[29]^ As the healthcare industry rapidly embraces simulation, it will increasingly become more dependent on simulation. The 2019 IOM report claims that within the last 30 years, simulation has been transformative. The IOM report states that simulation has led to a reduction in medical errors. According to this report, 60%–90% of preventable deaths from medical errors were avoided due to the use of simulation-based programs.^[11]^ The IOM went further to state that the major risks to patients are now largely systems-based deficiencies. “The IOM calls for greater use of simulation for systems processes given its widespread success at the individual and team levels.”^[11]^

Simulation is in the process of moving to hybrid models that incorporate manikins or actors while using high-fidelity virtual reality models and augmented reality. SBT is also being utilized for interprofessional teamwork to improve patient care. Rather than simply using SBT to improve individual provider skillsets, it addresses the bigger picture of patient care from a teamwork perspective. The future of the simulation will be incorporated into employee evaluations and assessments. It may also find its way into orientation processes when hiring new employees. One significant aspect that must be investigated in future studies is the benefit that SBT has on patient care from individual healthcare professionals to interprofessional simulation training.

In addition to the clinical SBT applications discussed throughout this paper, SBT has the potential to be extended to healthcare operations and management. According to Barjis,^[30]^ simulation should be “incorporated as a component of ongoing efforts to monitor and improve performance and increase efficiency by being incorporated into overall systems that enable an organization to operate.” This simulation model aims to adapt to changes as the system operates and collects data to track progress. Moving forward, Barjis^[30]^ suggests that organizations benefit from simulation models that are integrated into all areas of healthcare. Barjis^[30]^ explains that the objective “is not to treat simulation as a tool for conducting a one-time set of experiments when a major change is planned,” instead it is to “make the simulation models run in parallel to other applications as a routine part of the everyday work environment.” The current and future purpose of simulation is to make healthcare delivery safer and more efficient.

## 8 Conclusion

Historically and presently, simulations have demonstrated the ability to imitate real situations or scenarios in interactive environments.^[31]^ Throughout history, the basic principles of simulation center around the opportunity to educate participants and eventually achieve mastery in a safe environment. “Simulation-mediated education offers learners a safe practice environment where they can engage in critical thinking without endangering themselves or others who might otherwise be subject to harm in a real-life event.”^[32]^ Despite the profound benefits, SBT is underutilized today due to factors, including an insufficient understanding of the types of SBT available, the conditions under which they can best be utilized, and how they can benefit key stakeholders, including healthcare organizations and the patients they serve. However, reflecting on their past evolution, current SBT technology, and the many benefits of their use may provide both fuel and a foundation for expanded use for SBT in the future; and with it, a safer and perhaps more efficient healthcare industry.

## Author contributions

JB was responsible for the conception of the work, design of the work, design of figures, acquisition of information, analysis of information, interpretation of information, drafting the work, revising the work critically for important intellectual content, final approval of the version to be published, and agreement to be accountable for all aspects of the work in ensuring that questions related to the accuracy or integrity of any part of the work are appropriately investigated and resolved.

AH was responsible for the conception of the work, design of the work, analysis of information, interpretation of information, drafting the work, revising the work critically for important intellectual content, final approval of the version to be published, and agreement to be accountable for all aspects of the work in ensuring that questions related to the accuracy or integrity of any part of the work are appropriately investigated and resolved.
